# Effects of Sizing Agents and Resin-Formulated Matrices with Varying Stiffness–Toughness Ratios on the Properties of Carbon Fiber Epoxy Resin Composites

**DOI:** 10.3390/polym16233447

**Published:** 2024-12-09

**Authors:** Pengfei Song, Qianli Fang, Wen Liu, Xinyue Ma, Qingchao Li, Mehraj-ud-din Naik, Mudasir Ahmad, Guoqing Huang, Chuncai Yang

**Affiliations:** 1 Institute of Catalysis for Energy and Environment, College of Chemistry and Chemical Engineering, Shenyang Normal University, Shenyang 110136, China; 18698785870@163.com (P.S.); 17863521569@163.com (Q.F.); 18241394119@163.com (W.L.); 15848894876@163.com (X.M.); 15945315056@163.com (Q.L.); 2 Department of Chemical Engineering, College of Engineering and Computer Science, Jazan University, Jazan 45142, Saudi Arabia; mnaik@jazanu.edu.sa; 3 School of Chemistry and Chemical Engineering, Northwestern Polytechnical University, Xi’an 710072, China; mirmudasirv@nwpu.edu.cn; 4 Jilin Qianren Innovative Materials Co., Ltd., Jilin 132101, China

**Keywords:** carbon fiber composite, resin matrix, interlaminar share strength, compressive strength

## Abstract

Interlaminar shear strength (ILSS) and compressive strength are two of the most critical properties of carbon fiber-reinforced polymer (CFRP). In this report, three types of epoxy resins—4,4’-diaminodiphenylmethane epoxy resin (AG-80), bisphenol A epoxy resin (E-1NT), and novolac epoxy (EPN)—were studied. E-1NT is characterized by low viscosity and low cost but exhibits poor mechanical properties, while AG-80 offers better wetting with carbon fiber. These two epoxy resins were mixed in various mass ratios. The study revealed that as the AG-80 content increased, the ILSS of the composite also increased, reaching a maximum of 94.04 MPa when the AG-80 content reached 60%. Beyond this point, further increases in AG-80 did not enhance the ILSS. Conversely, the compressive strength initially increased but then declined sharply as the AG-80 ratio increased. The maximum compressive strength was recorded at 748.52 MPa when the AG-80 content reached 60%, which was 21% higher than pure AG-80 and 32% higher than pure E-1NT. Additionally, the study examined three different types of ionic sizing agents and four different resin matrices (E-1NT/DDS, AG-80/DDS, AG-80/E-1NT/DDS, EPN/DDS). Among them, the 60% AG-80/40% E-1NT/DDS/CF formulation demonstrated the best balance in both ILSS and compressive strength.

## 1. Introduction

Carbon fiber-reinforced resin composites possess outstanding properties, including being lightweight, having a high specific strength, a high specific modulus, and excellent fatigue durability [1,2,3]. They are widely used in various fields, including transportation, aerospace, energy, and many others [4,5,6]. As one of the most commonly used resin matrices, epoxy resin has several advantages, including excellent adhesion, heat resistance, chemical resistance [7,8,9], high strength, and more. These properties significantly enhance the performance of carbon fiber composite materials [1]. AG-80 is a kind of high-performance EP; its advantages are its high heat resistance and excellent chemical resistance, and the cured resin exhibits good heat resistance and chemical stability [10,11,12]; while bisphenol A epoxy resin is most common and cheap EP, its ether bond (-O-) and carbon bond (C-C) have flexible cross-linked macromolecules [13], and its ether bond and hydroxyl are polar groups, which help to improve wettability and adhesion [14,15,16]. Bisphenol A epoxy resin was added to AG-80, which improved the specific properties of CFRP [12]. However, studies of the effect of the viscosity and crosslinking density of the formulated mixing EP of AG-80 and E-1NT on ILSS and the compressive strength of CF/EP composites are rare.

The mechanical properties of CFRP depend not only on the properties of the reinforcing fiber and resin matrix [17,18,19,20,21], but also on the interface properties of the fiber/sizing agent and sizing agent/matrix [22,23]. The commercial CF was produced through the carbonization [24] and graphitization process of the stacked inert carbon microcrystals making the CF surface smooth, chemically inert, and with poor wettability; therefore, CF’s surface was required to be modified by the sizing agent. The sizing agents can not only improve the surface wettability of the CF but also reduce the fluffs of fiber bundles and protect the CF from external mechanical damage. There are many studies on the effect of sizing agents on the interface of carbon fiber and ILSS of composites [25], but there are no reports on a systemic study of the influence of types of sizing agents on the mechanical properties of composites.

In this report, three types of sizing agents—cationic (S1), non-ionic (S2), and anionic (S3)—were used to coat the CF and their effects on the ILSS and compressive strength of the CF/EP composite were studied. The effects of resin matrix and their crosslinking density on the mechanical properties of CF/EP were another focus research in this paper; thus two types of epoxy resins, AG-80 and bisphenol A epoxy resin of E-1NT, were formulated by mass ratio and then reacted with the curing agent of DDS [26], and the crosslinking density of the mixing resin matrixes became lower than that of the 100% AG-80 [27]. The effects of the crosslinking density of the mixing resin matrixes on the ILSS and compressive strength of CFRP were studied. At the same time, the synergistical effects of both the sizing agents and resin matrix on the mechanical properties of the CF/EP composite were investigated; thus, three types of sizing agents (cationic S1, non-ionic S2, anionic S3) and four different resin matrices (E-1NT/DDS, AG-80/DDS, 60% AG-80/40%E-1NT/DDS, EPN/DDS) were also studied [28,29,30], systematically. The maximum compressive strength was reached when AG-80 and E-1NT were 60% and 40%, respectively, with the curing sizing being DDS, and the sizing agent being cationic-type S1.

## 2. Experiment

### 2.1. Material

Here, T400 carbon fiber tow was sized using three different sizing agents: S2, obtained from the Toray Co., Japan, and S1 and S3, supplied by the Jilin Qianren Innovative Materials Co. Ltd. (Jilin, China), with their compositions remaining proprietary. E-1NT, with a molecular weight of 370 and an epoxy equivalent of 185, was also provided by the Jilin Qianren Innovative Materials Co. Ltd. AG-80, a 4,4’-diaminodiphenylmethane epoxy resin with an epoxy equivalent of 114, and the curing agent DDS (4,4’-diaminodiphenyl sulfone) were supplied by the Tiantai High-tech Co. Ltd. (Guangzhou, China). Additionally, EPN, with an epoxy equivalent of 174.6, was sourced from the Nanya Electronic Materials (Kunshan) Co. Ltd. (Jiangsu, China).

### 2.2. Specimen Preparation

**Figure 1:**
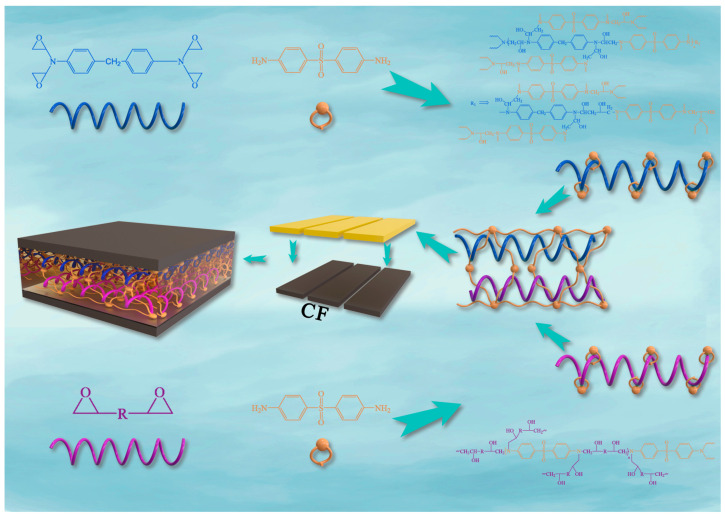
Preparation of the AG-80/E-1NT/DDS/CF composite.

**Figure 2:**
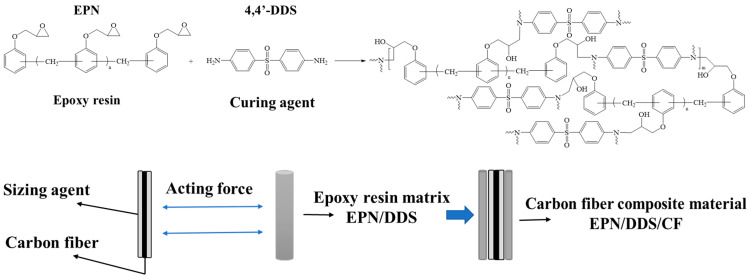
Preparation of the CF/EPN/DDS composite.

The unidirectional carbon fiber/epoxy (CF/EP) composite specimens were prepared using a two-stage curing process, with a carbon fiber volume fraction of approximately 65%. The first stage involved pre-curing at 135 °C. At this temperature, the viscosity of the formulated composition of epoxy resins and the curing agent was low, resulting in a long gelling time, which facilitated the wetting and penetration of the carbon fiber bundles. The second stage completed the curing process at 180 °C. The three types of epoxy resins (AG-80, E-1NT, and EPN) and the formulated resin matrices of AG-80/E-1NT, mixed in various ratios, were cured using the curing agent DDS, as illustrated in Figure 1 and Figure 2.

### 2.3. Characterization

Briefly, 100 g of AG-80, E-1NT, or a mixture of AG-80 and E-1NT with 40 g of DDS was prepared in a beaker and pretreated at 135 °C. The formulation ratio of AG-80 to E-1NT was varied from 0% to 100%. The viscosity was measured and recorded every 10 min using a Brookfield dial viscometer (LVT230), and the test was halted when the viscosity approached 2000 cP.

**Figure 3:**
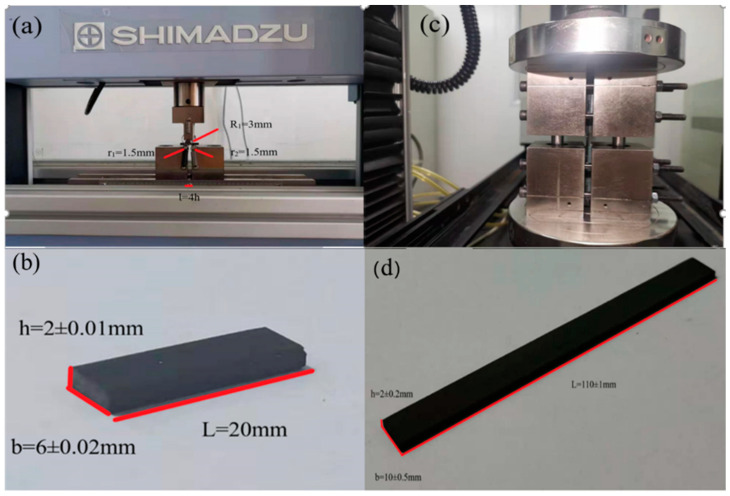
The instrument (**a**) and specimen size (**b**) in testing ILSS; the instrument (**c**) and specimen size (**d**) in testing compressive strength.

Interlaminar shear strength (ILSS) tests were conducted using a universal testing machine (AGS-X10KN, Shimadzu, Tokyo, Japan) in accordance with GB/T 30969-2014. The loading speed of the crossbeam was set at 1 mm/min until the maximum failure load of the sample was recorded. The testing apparatus is shown in Figure 3a, while the dimensions of the test specimens are presented in Figure 3b. The results were derived from the average values of at least seven specimens tested for each type of composite. The ILSS values were calculated using the following equation:

(1) $$\mathrm{ILSS}=\frac{3P\;}{4bh}$$

where *P* is the maximum load in newtons (N), *b* is the width of the test specimen in millimeters (mm), and *h* is the thickness of the test specimen in millimeters (mm).

Compressive strength tests were also performed using a universal testing machine (Instron-5869, Instron Corporation, Canton, MA, USA) in accordance with GB/T 5258-2008 [31]. The loading speed of the crossbeam was set at 3 mm/min until the maximum failure load of the sample was recorded. The testing apparatus is shown in Figure 3c, and the dimensions of the test specimens are displayed in Figure 3d. The results were calculated based on the average values of at least seven specimens tested for each type of composite. The compressive strength (*σcM*) and compression modulus (*E_c_*) were determined using the following equation:

(2) $$\sigma cM=\frac{F}{bh}$$

where *F* is the maximum load in newtons (N), *b* is the width of the test specimen in millimeters (mm), and *h* is the thickness of the test specimen in millimeters (mm).

Fracture images of the carbon fiber composite were examined using a scanning electron microscope (SEM) (JSM-7610F Plus, JEOL, Akishima, Japan)

## 3. Results and Discussion

### 3.1. Resin Matrix Viscosity Test

**Figure 4:**
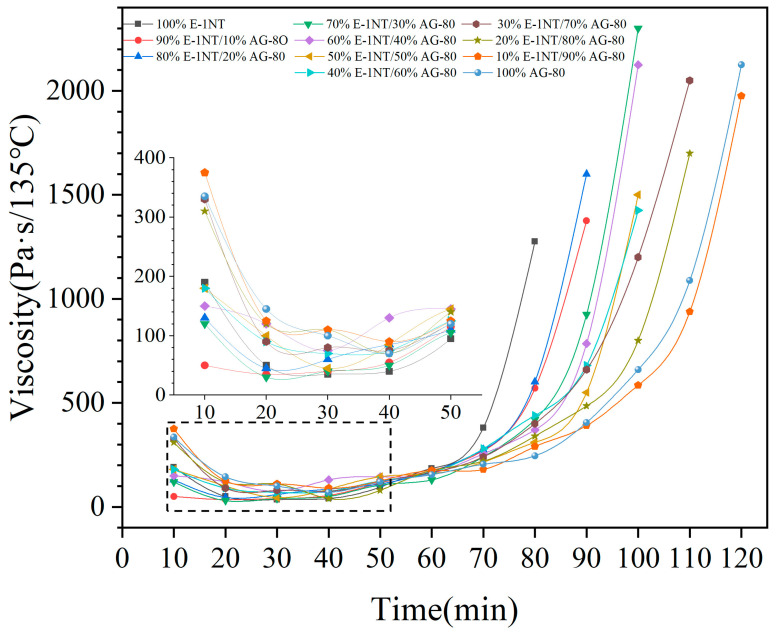
Viscosity changes vs. time.

Resin matrix viscosity has a huge effect on wetting the carbon fiber interface and penetration inside the fiber tow [32], so it is necessary to test the viscosity of the formulated composition of the epoxy resin and its curing agents at different temperatures. As shown in Figure 4, E-1NT/DDS decreased and took 30 min to reach its lowest viscosity and then increased to nearly 2000cPs in 80 min at 135 °C; correspondingly, AG-80/DDS decreased and took 40 min to reach its lowest viscosity and then increased to nearly 2000 cPs in 120 min at 135 °C. The gel time of AG-80/DDS was longer than that of E-1NT/DDS. After AG-80/DDS or E-1NT/DDS was pretreated at 135 °C for 40 min or 30 min, the carbon fiber tow was laminated with the resin matrix at its lowest viscosity. The resin matrix facilitated its wetting and adhesion to the carbon fiber tow when the viscosity was lowest [25]. The gel time of the formulated composition of AG-80/E-1NT/DDS was shorter than that of AG-80/DDS and longer than that of E-1NT/DDS.

### 3.2. ILSS and Compression Strength Test of AG-80/E-1NT/DDS/CF

**Figure 5:**
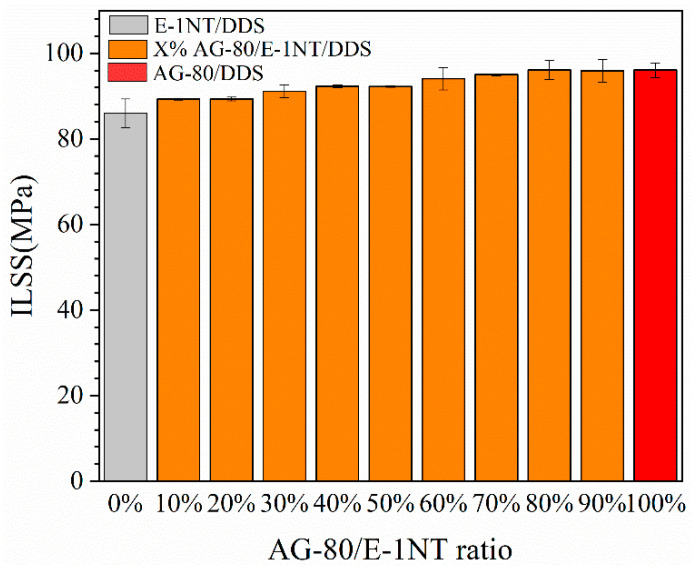
ILSS of the CF composites reinforced with different ratios of AG-80/E-1NT/DDS.

Interlaminar shear strength (ILSS) is a common parameter for evaluating interfacial adhesion in epoxy resin/carbon fiber composites [33]. The interfacial adhesion between the resin matrix and fiber bundle can be visually observed by SEM. It can be seen from the reaction equation in Figure 1 that AG-80 had more epoxy groups than E-1NT; the epoxy value (epoxy value of 0.85) of AG-80 was bigger than that of E-1NT (epoxy value of 0.56), and the crosslinking density of AG-80/DDS was higher than that of E-1NT/DDS. Therefore, the crosslinking density of the mixture of AG-80/E-1NT/DDS increased as the AG-80 ratio increased. In Figure 5, the ILSS of the E-1NT /DDS/CF composite was 86 MPa, while the ILSS of the AG-80/DDS/CF composite was 96.09 MPa. When the ratio of AG-80 was between 0% and 80%, the ILSS of the AG-80/E-1NT/DDS/CF composite increased with AG-80; when AG-80 increased to 80%, the ILSS of AG-80/E-1NT/DDS/CF composite reached a maximum value of 96.09 MPa. When the ratio of AG-80 increased from 80% to 100%, the ILSS of AG-80/E-1NT/DDS/CF composite did not increase further.

**Figure 6:**
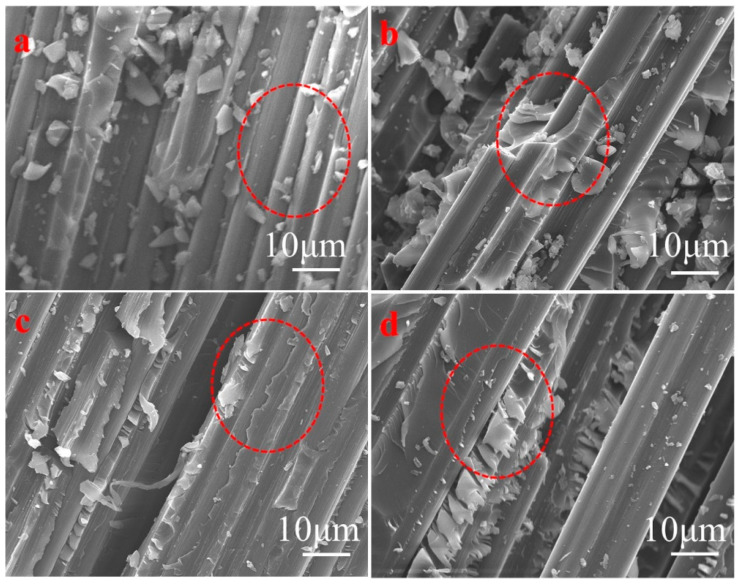
SEM images of fracture surface in the CF/EP composites: (**a**) 100% E-1NT/DDS/CF; (**b**) 20% AG-80/80% E-1NT/DDS/CF; (**c**) 60% AG-80/40% E-1NT/DDS/CF; (**d**) 100% AG-80/DDS/CF.

The composite of 100% E-1NT/DDS/CF and 20% AG-80/80% E-1NT/DDS, as shown in Figure 6a, b, has many small resin non-bonded fragments on the CF surface, and the carbon fiber tow was exposed to the damage on the interface, indicating poor bonding ability between the resin matrix and the carbon fiber, which was consistent with the ILSS. AG-80 has tertiary amino groups, and CF has carboxyl groups, forming a positive–negative charge interaction between the resin matrix and CF, so that the wetting and adhesion between the CF and resin matrix increased with AG-80 in the AG-80/E-1NT/DDS/CF composite. It can be seen that the carbon fiber tow is still wrapped in a large amount of resin, and only a few small-sized resin fragments have fallen off onto the surface, in the fracture images of the composite of 60% AG-80/40% E-1NT/DDS/CF and AG-80/DDS/CF, as shown in Figure 6c,d, supporting the theory that their ILSS increased, as discussed in the above Section.

**Figure 7:**
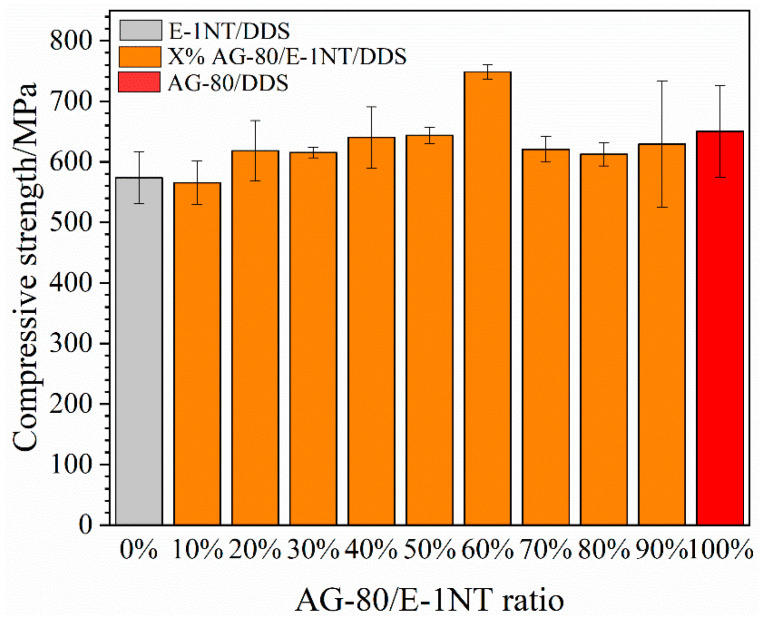
Compressive strength with different ratios of AG-80 among AG-80/E-1NT/DDS/CF.

Compressive strength is the most important property of the CF composite that affects its application [34]. AG-80 has four epoxy groups, E-1NT has two epoxy groups with a relatively lower crosslinking density that reacts with the curing agent of DDS, and the formulated resin matrix of 60% AG-80 and 40%E-1NT is between the two. The crosslinking density of the resin matrix can affect the compression strength of the CF composite. As shown in Figure 7, the compression strength of E-1NT/DDS/CF was 573.32 MPa. As the content of AG-80 increased from 0% to 60%, the crosslinking density of the formulated resin matrix increased, and the compressive strength increased; the composite of 60% AG-80/40% E-1NT/DDS/CF had the highest compressive strength, but as AG-80 increased further from 60% to 100%, the compressive strength decreased first and then fluctuated.

**Figure 8:**
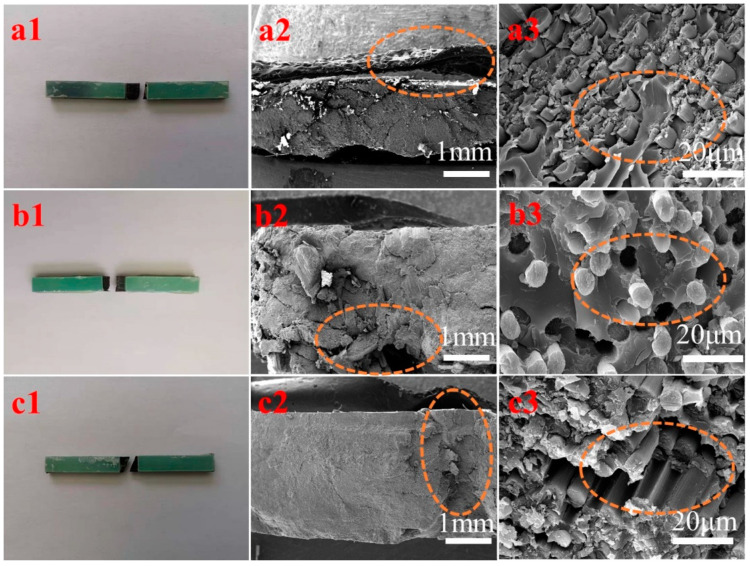
SEM images of the CFRP cross-section (compressive strength test): (**a1**–**a3**) 60% AG-80/40% E-1NT/DDS/CF; (**b1**–**b3**) AG-80/DDS/CF; (**c1**–**c3**) E-1NT/DDS/CF.

The surface morphology and fracture mode of the CF composite are related to its compressive strength. Figure 8(a1,b1,c1) show the CFRP fracture mode in the middle meeting with the compressive strength test requirement. Figure 8(a2) had a smooth fractured section, whereas Figure 8(b2,c2) did not have a fractured section. AG-80/DDS/CF shows an interface shear fracture (Figure 8(b3)), E-1NT/DDS/CF has a layered fracture (Figure 8(c3)), and 60% AG-80/40% E-1NT/DDS/CF has an interface pull-out fracture (Figure 8(a3)), with the highest compression strength at 748.52 MPa (as shown in Figure 7).

In addition to the effect of the above-mentioned crosslinking density, there were other factors such as interfacial wetting and adhesion between the CF and the resin matrix involved. AG-80 forms positive and negative charges with the CF, which is beneficial for improving the compressive strength. As the proportion of AG-80 in the formulated resin increases, the compressive strength of the composite also increases. However, due to the relatively high viscosity of AG-80, the wettability between the resin matrix and fibers and the penetration inside the fiber tow deteriorates, which affects the fracture mode of the composite material (as shown in Figure 8(a3,b3,c3)). E-1NT resin has a relatively lower viscosity than AG-80; therefore, due to this low viscosity, the formulated resin matrix of 60% AG-80 and 40% E-1NT was conducive to resin infiltration inside the carbon fiber interface and fiber tow. At the same time, the formulated resin matrix forms a positive and negative charge to increase the wetting and adhesion between the fibers and resin matrix due to the existing AG-80.

Therefore, the fibers are fully filled by the formulated resin matrix of AG-80 and E-1NT, and the fracture mode of the composite material was an interface pull-out fracture (as shown in Figure 8(a3)), which had the highest compressive strength (as shown in Figure 7). Due to the higher crosslinking density of AG-80/DDS, the fracture mode of the AG-80/DDS/CF composite was the delamination of an interface shear fracture (as shown in Figure 8(b3)), with its compressive strength reduced. Finally, the E-1NT resin lacks positive and negative charges at the interface with the carbon fibers, resulting in poor bonding between the resin matrix and fibers. The fracture mode of the E-1NT/CF composite material is a pull-out layered fracture (as shown in Figure 8(c3)), and its compressive strength is also affected.

### 3.3. The Effect of Sizing Agents on ILSS and Compressive Strength of CF Composite

Sizing agents were the most important surface modification additives and affected the interfacial wetting and adhesion between the CF and the resin matrix, which, in turn, affected the compressive strength of the CF composite. Three kinds of sizing agents, S1, S2, and S3, were studied and analyzed by acid-base titration; the results are shown in Table 1. S1 is a cationic sizing agent, S2 is a non-ionic sizing agent, and S3 is an anionic sizing agent.

**Figure 9:**
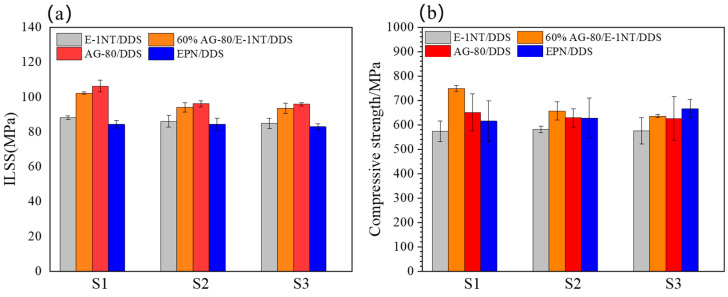
(**a**) ILSS with four types of resin matrix/sizing agents S1, S2, and S3/CF. (**b**) Compressive strength with four types of resin matrix/sizing agents S1, S2, and S3/CF.

**Figure 10:**
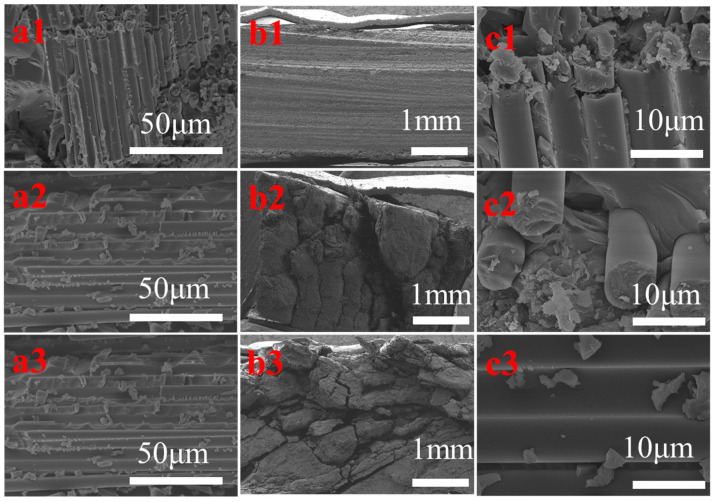
Photo and SEM images of the CFRP cross-section: (**a1**) the section of 60% AG-80/40% E-1NT/DDS/S1/CF (ILSS test); (**a2**) the section of 60% AG-80/40% E-1NT/DDS/S2/CF (ILSS test); (**a3**) the section of 60% AG-80/40% E-1NT/DDS/S3/CF (ILSS test); (**b1**) the section of 60% AG-8O/40% E-1NT/DDS/S1/CF (compressive strength test); (**b2**) the section of 60% AG-8O/40% E-1NT/DDS/S2/CF (compressive strength test); (**b3**) the section of 60% AG-8O/40% E-1NT/DDS/S3/CF (compressive strength test); (**c1**) the section of 60% AG-80/40% E-1NT/DDS/S1/CF (compressive strength test); (**c2**) the section of AG-80/DDS/S1/CF (compressive strength test); (**c3**) the section of E-1NT/DDS/S1/CF (compressive strength test).

Different types of sizing agents affect the ILSS of carbon fiber composites. Figure 9a shows that the sizing agents S1, S2, and S3 had an effect on the ILSS of the CF composite, but for different resin matrixes and formulated resin matrixes, the effect trend was the same. The cationic sizing agent S1 had a tertiary amino group and could form positive and negative charges with the CF, in turn increasing the interfacial adhesion between the carbon fiber and sizing agents. Therefore, the cationic sizing agent S1 exhibited the highest ILSS, the non-ionic sizing agent S2 showed the next best strength ILSS, and the anionic sizing agent S3 showed the weakest ILSS. Figure 10(a1–a3) are SEM fracture images of composites after their ILSS testing. It can be seen from Figure 10(a1) that there are a large number of resin fragments between fibers, while Figure 10(a2,a3) have fewer resin fragments exposed on the surface of the fiber bundle compared to Figure 10(a1), consistent with their ILSS.

The ionic types of sizing agents may affect the compressive strength of carbon fiber composites. Figure 9b shows that the sizing agents S1, S2, and S3 affected the compressive strength of the carbon fiber composite. Compared with the sizing agents S2 and S3, the cationic type of sizing agent S1 had the highest compressive strength of 748.52 MPa. Besides the effect of the ionic type of sizing agents on the compressive strength of carbon fiber composites, the resin matrix also influenced the compressive strength. As for the sizing agents S1 and S2, the composite of 60% AG-80/40% E-1NT/DDS/CF had the highest compressive strength of 748.52 MPa and 656.57 MPa, respectively. However, for the sizing agent S3, the composite of EPN/DDS/CF had the highest compressive strength of 665.75 MPa. As for the formulated resin matrixes of AG-80 and E-1NT, the composite of 60% AG-80/40% E-1NT/DDS had the highest compressive strength, which is consistent with the results discussed in Figure 7.

Figure 10(b1–b3) show the effect of sizing agents S1, S2, and S3 on the fracture surface of the composite of 60% AG-80/40% E-1NT/DDS/CF. The sized CF composite by the cationic sizing agent S1 shows a relatively flat and smooth fracture surface (Figure 10(b1)), indicating that the resin matrix had strong adhesion with CF, while the CF composite by the non-ionic sizing agent S2 shows a rough fracture surface containing a small number of gaps. Figure 10(b2) indicates that the resin matrix has decreasing adhesion with the CF, and the CF composite by the anionic sizing agent S3 shows a very rough fracture surface containing a large number of gaps. Figure 10(b3) indicates that the resin matrix has the weakest adhesion with the CF, so the cationic sizing agent of S1 enhances the compressive strength of the composite due to the increasing interfacial adhesion between the CF and the sizing agent S1 by positive and negative charge interactions.

Figure 10(c1–c3) show the effect of the formulated resin matrix with various ratios of AG-80 and E-1NT on the fracture surface of the composite. The sizing agent was cationic-type S1, the fracture surface of 60% AG-80/40% E-1NT/DD/CF was filled with a large amount of resin indicating strong wetting behavior (Figure 10(c1)), while the fracture surface of AG-80/DD/CF had a small number of gaps unfilled by resin, indicating decreasing wetting behavior (Figure 10(c2)), and the fracture surface of E-1NT/DD/CF had almost no residual resin on the surface (Figure 10(c3)), further indicating that its wetting behavior is weakest.

## 4. Conclusions

In this research, the effects of three kinds of epoxy resins and their formulating compositions, and three ionic types of sizing agents on the ILSS and compressive strengths of EP/CF composites, were studied. Different ratios of AG-80 and E-1NT epoxy resin were formulated. The AG-80 epoxy resin matrix has tertiary amino groups and forms positive–negative charge interactions with the carboxyl group of the CF, which increases the adhesion between AG-80 and the CF, resulting in the highest ILSS, no matter which ionic type, S1, S2, or S3, was used, with the maximum ILSS of AG-80/DDS/CF reaching 96.09 MPa. In contrast, the compressive strength of the composite was influenced by various factors such as the ionic type of the sizing agents, crosslinking density, and the viscosity of epoxy-formulating compositions. The results showed that the cationic-type sizing agent S1 was able to increase the compressive strength of EP/CF due to the positive–negative charge interaction of its tertiary amino groups with the carboxyl group of the CF, with the composite of 60% AG-80/40% E-1NT/DDS/CF having the highest compressive strength of 748.52 MPa among the various ratios of the formulating composition of AG-80 and E-1NT, which is consistent with its lowest viscosity at the precuring stage of 135 °C and appropriate crosslinking density, with its compressive strength respectively increasing by 14% and 18% compared to the non-ionic-type sizing agent S2 and anionic-type sizing agent S3. However, for the anionic-type sizing agent S3, among AG-80, E-1NT, and various ratios of their mixing composition, the interaction between the CF and epoxy resin looked negligible, with the composite of EPN/DDS/CF showing the highest compressive strength of 665.75 MPa.

## Figures and Tables

**Table 1:** Ionic test results of three different types of sizing agents.

Numbering	Acetic Acid	Sodium Hydroxide Solution	Type
S1	No precipitation	Precipitation	Cationic sizing agent
S2	No precipitation	No precipitation	Non-ionic sizing agent
S3	Precipitation	No precipitation	Anionic sizing agent

## Data Availability

The original contributions presented in this study are included in the article/Supplementary Materials. Further inquiries can be directed to the corresponding authors.

## Supplementary Materials

The following supporting information can be downloaded at: https://www.mdpi.com/article/10.3390/polym16233447/s1.
