# Supplementary material for: Effects of Sizing Agents and Resin-Formulated Matrices with Varying Stiffness–Toughness Ratios on the Properties of Carbon Fiber Epoxy Resin Composites
Source: Polymers (Basel). 2024 Dec 9;16(23):3447. doi: 10.3390/polym16233447 (PMC11644070; doi:10.3390/polym16233447)
Supplement: Supplementary file 1 [file polymers-16-03447-s001.zip › polymers-3332013-supplementary.pdf]

T1

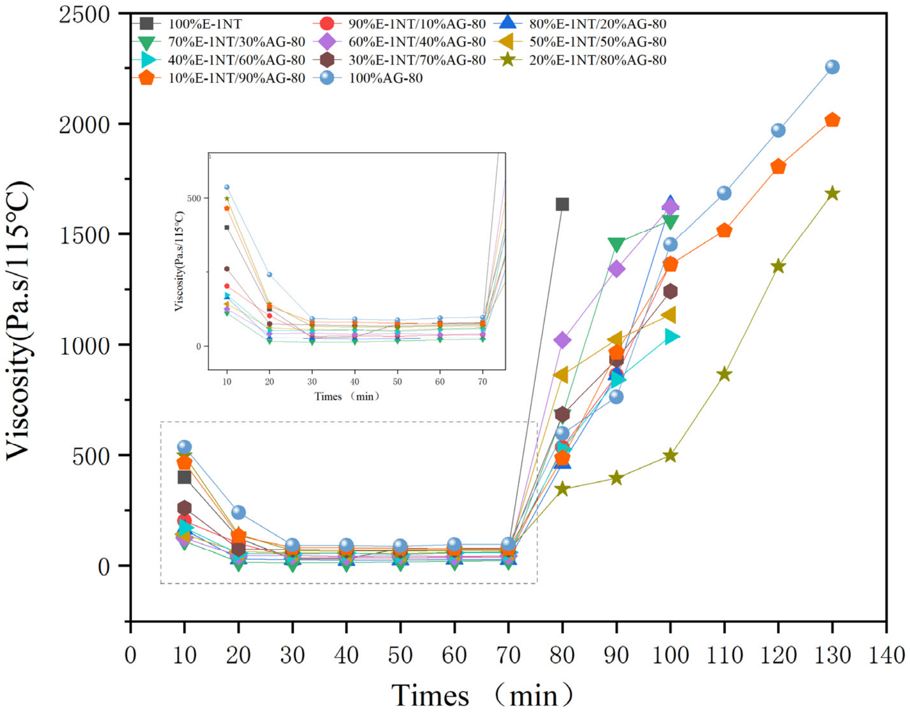

Viscosity changes vs. time.

T2

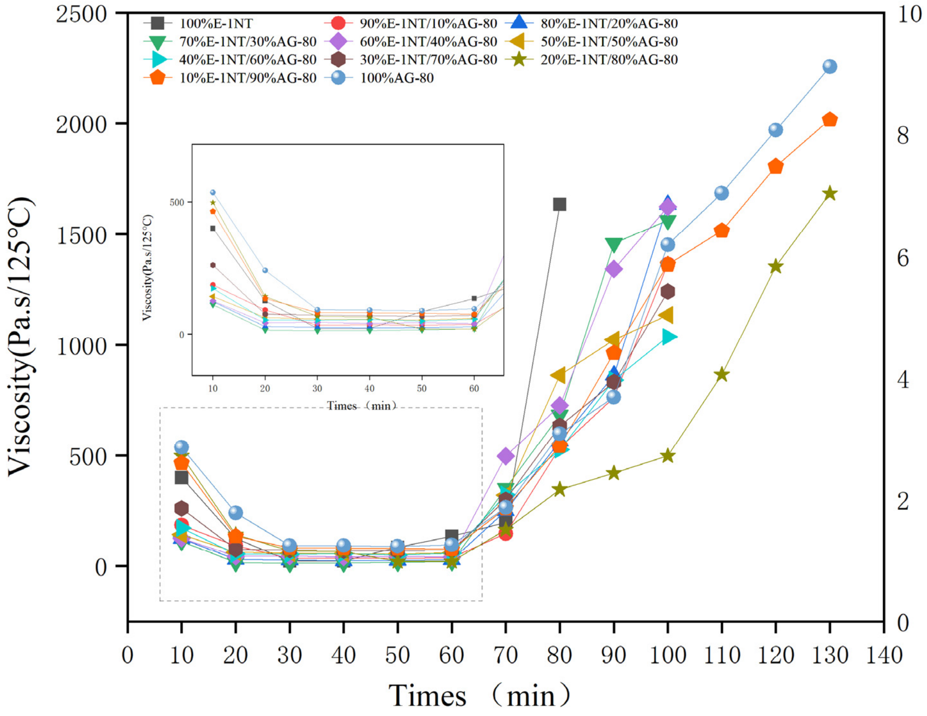

Viscosity changes vs. time.

T3

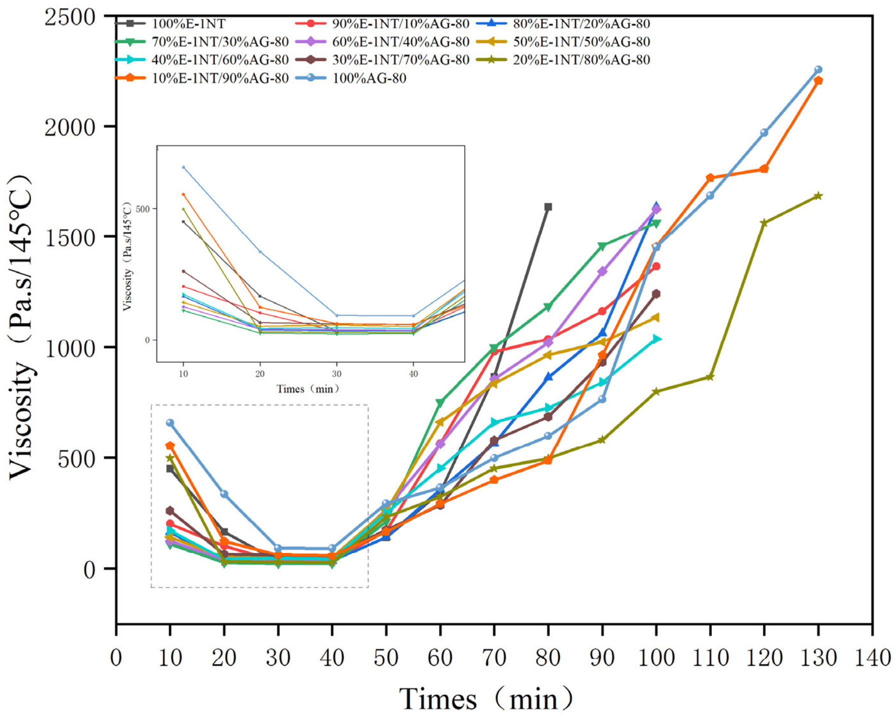

Viscosity changes vs. time.

T4

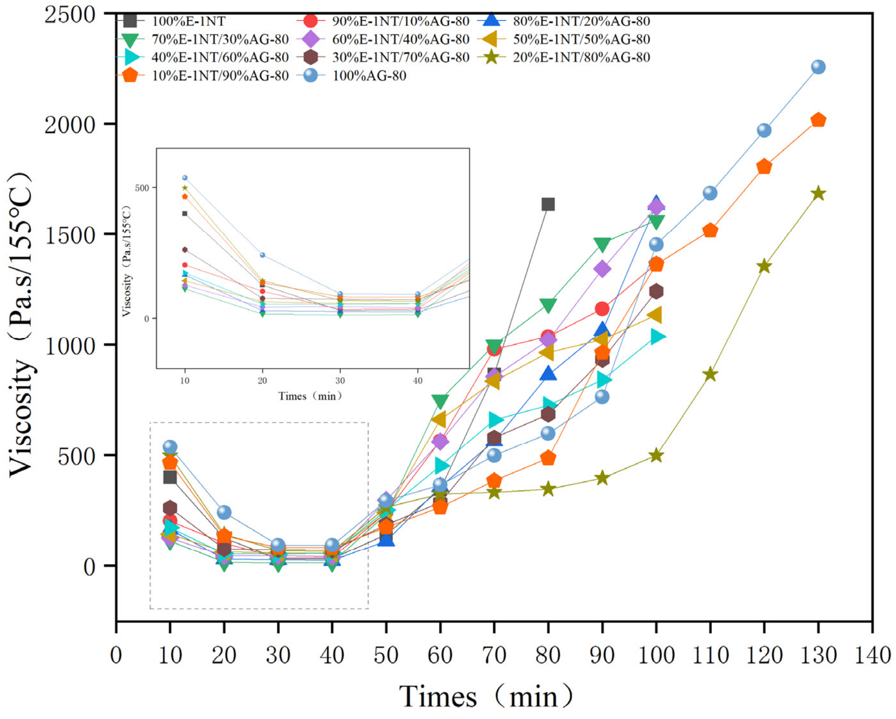

Viscosity changes vs. time.
